# Supplementary material for: Topical Imiquimod in Primary Cutaneous Extramammary Paget’s Disease: A Systematic Review
Source: Cancers (Basel). 2023 Nov 30;15(23):5665. doi: 10.3390/cancers15235665 (PMC10705770; doi:10.3390/cancers15235665)
Supplement: Supplementary file 1 [file cancers-15-05665-s001.zip › cancers-2739846-supplementary.pdf]

Supplementary material

List of supplementary materials:

1. **Supplementary Table S1:** PRISMA checklist.
2. **Supplementary Table S2:** Main characteristics of the included studies and summery of demographics and EMPD features.
3. **Supplementary Table S3:** Results for JBI critical appraisal tool for quasi-experimental studies.
4. **Supplementary Table S4:** Results for JBI critical appraisal tool for case series studies.
5. **Supplementary Table S5:** Results for JBI critical appraisal tool for case report studies.

Table S1: PRISMA checklist.

Section/topic	#	Checklist item	Reported on page #
**TITLE**			
Title	1	**Topical Imiquimod in primary cutaneous extramammary Paget's disease: a systematic review.**	1
**ABSTRACT**			
Structured summary	2	Provide a structured summary including, as applicable: background; objectives; data sources; study eligibility criteria, participants, and interventions; study appraisal and synthesis methods; results; limitations; conclusions and implications of key findings; systematic review registration number.	1
**INTRODUCTION**			

Rationale	3	Describe the rationale for the review in the context of what is already known.	2
Objectives	4	Provide an explicit statement of questions being addressed with reference to participants, interventions, comparisons, outcomes, and study design (PICOS).	2
**METHODS**			
Protocol and registration	5	Indicate if a review protocol exists, if and where it can be accessed (e.g., Web address), and, if available, provide registration information including registration number.	2
Eligibility criteria	6	Specify study characteristics (e.g., PICOS, length of follow-up) and report characteristics (e.g., years considered, language, publication status) used as criteria for eligibility, giving rationale.	2-3
Information sources	7	Describe all information sources (e.g., databases with dates of coverage, contact with study authors to identify additional studies) in the search and date last searched.	3
Search	8	Present full electronic search strategy for at least one database, including any limits used, such that it could be repeated.	3
Study selection	9	State the process for selecting studies (i.e., screening, eligibility, included in systematic review, and, if applicable, included in the meta-analysis).	4
Data collection process	10	Describe method of data extraction from reports (e.g., piloted forms, independently, in duplicate) and any processes for obtaining and confirming data from investigators.	3
Data items	11	List and define all variables for which data were sought (e.g., PICOS, funding sources) and any assumptions and simplifications made.	3
Risk of bias in individual studies	12	Describe methods used for assessing risk of bias of individual studies (including specification of whether this was done at the study or outcome level), and how this information is to be used in any data synthesis.	3
Summary measures	13	State the principal summary measures (e.g., risk ratio, difference in means).	3
Synthesis of results	14	Describe the methods of handling data and combining results of studies, if done, including measures of consistency (e.g., I^2) for each meta-analysis.	3

Table S2. Main characteristics of the included studies and summery of demographics and EMPD features.

Nº	Author	Year of publication	Study design	LE	Conflict of interest	Nº of Participant	Nº of cases	Age (years) Md (M)	Age (years) Range (SD)	Sex (F/Ml)	Race	Nº of lesions	Lesion location	Tumor size Md (M)	Tumor size Range (SD)	Tumor stage	Duration of EMPD (months) Md (M)	Duration of EMPD (months) Range (SD)
1 [19]	Zampogna, J.C.	2002	Case report	4	Declared	2	2	57		Ml	Caucasian (2p)	1	Scrotum	3x5		0 (2p)	48	
								72		Ml		2	Perineal and inguinal	NR			120	
2 [20]	Mirer, E	2006	Case report	4	Not available	2	2	80		F	Caucasian (2p)	1	Intergluteal fold, perianal and vulva	6x9		0 (2p)	5	
								90		F		1	Nipple and areola	10			180	
3 [36]	Hatch, K.D.	2008	Case report	4	Not available	2	2	68		F	NR	1	Vulva and perianal	NR		0 (2p)	72	
								60		F		1	Vulva, groin, buttocks, and upper thigh				156	
4 [38]	Challenor, R.	2009	Case report	4	None declared	2	2	48		F	Caucasian (2p)	1	Vulva	NR		0 (2p)	12	
								66		F		1	Vulva				24	
5 [32]	Tanaka, V.D.A.	2009	Case series	4	Not available	14	1	68		M	Caucasian	1	Axilla	8		0	5.4[1]	1-15[1]
6 [5]	Pang, J.	2010	Case series	4	None declared	14	12	(73)[1]	58-91[1]	F (12p), Ml (2p)[1]	NR	15[1]	Axilla (1p) and genital area (13p)[1]	(6.7)[1]	2-15[1]	0 (12p)	34.7[1]	0-74[1]
7 [34]	Sendagorta, E.	2010	Case report	4	None declared	3	3	66	58-82	F (3p)	Caucasian (3p)	1	Vulva (3p)	NR		0 (3p)	6	2-12
8 [42]	Shelbi, C.J.O.	2011	Case report	4	None declared	2	2	75		Ml (2p)	Asian (2p)	1	Scrotum	NR		0 (2p)	12	
								70				1	scrotum, groin and perineum				48	
9 [31]	Baiocchi, C.	2012	Case series	4	None declared	4	4	(62.2)	56-80	F (4p)	Caucasian (3p), Asian (1p)	4	Vulva (4p) and perianal (3p), perineal (3p)	NR		0 (4p)	NR	
10	Boulard, C.	2013	Case report	4		2	2	40		F (2p)		1	Vulva (2p)	NR		0 (2p)	NR	

[39]					None declared			61			Caucasian (2p)	1					
11 [1]	Choi, J.H.	2013	Case series	4	Not available	10	10	65.5	60-81	F (3p), Ml (7p)	Asian (10p)	10	penescrotal (5p), vulva (3p), inguinal (2p)	NR	0 (10p)	(73.6)	4-360
12 [14]	Sanderson, P.	2013	Case series	4	None declared	6	6	70	58-85	F (6p)	NR	6	Vulva (6p), perineal (2p), anus (1p), groin (1p), thigh (1p)	NR	0 (6p)	15	6-24
13 [40]	Jing, W.	2014	Case report	4	Not available	2	2	72		Ml (2p)	Asian (2p)	2	Scrotum, groins, perineum and penis	NR	0 (2p)	48	
								56				2	Scrotum, groin and penis			24	
14 [33]	Luyten, A.	2014	Case series	4	None declared	21	21	(66.4)	41-84	F (21p)	NR	21	Vulva (21p)	NR	0 (21p)	NR	
15 [34]	Marchitelli, C.	2014	Case series	4	None declared	10	10		60-92	F (10p)	NR	NR	Vulva (10p)	NR	0 (10p)	NR	
16 [27]	Cowan, R.A.	2016	Observational trial	3	None declared	8	8	71.5	47-78	F (8p)	Caucasian (7p), Asian (1p)	NR	vulva (8p) and perianal (1p)	NR	0 (8p)	NR	
17 [3]	Liau, M.M.	2016	Case series	4	None declared	6	7	74.5	66-83	F (4p), Ml (2p)	Asian (7p)	7	Vulva (3p), both axilla (1p), Scrotum (1p), Penile shaft (1p)	NR	0 (6p)		12-60
18 [29]	Sawada, M.	2018	Observational trial	3	None declared	9	9	82	61-94	F (5p), Ml (4p)	Asian (9p)	NR	vulva (5p), penile shaft (4p) and scrotum (1p)	NR	0 (9p)	NR	
19 [41]	Apalla, Z.	2018	Case report	4	None declared	2	2	48		F	NR	1	**genital** and **perianal**	NR	0 (2p)	NR	
								62		Ml		1	Pubic	2.5			
20 [30]	Van der Linden , M.	2019	Cohort	3	None declared	113	18	73[1]	41-97[1]	18 F	NR	NR	Vulva (18p)	NR	0 (18p)	NR	
21 [15]	Choi, S.	2021	Case series	4	None declared	166	25	(65.4)[1]	37–84[1]	F (44p), Ml (122 p)[1]	Asian (25p)	NR	scrotum, vulva, penile, perianal, other (nº NR)	NR	0 (25p)	NR	

22 [35]	Christodoulidou, M.	2021	Case series	4	None declared	10	5	71	66-82	Ml (5p)	NR	NR	inguinal (2) and penoscrotal (5)	NR		0 (5p)	NR	
23 [28]	Van der Linden , M.	2022	Clinical Trial	2	None declared	24	23	67	42-84	F (24p)	Caucasian (22p), Hispanic (1p), Asian (1p)	NR	Vulva (24p)	16 cm^2	3-130 cm^2	0 (24p)	24	0-216
24 [6]	Borella, F.	2022	Cohort	3	None declared	55	55	(63)	36–92 (± 12)	F (55p)	Caucasian (55p)	NR	Vulva (55p)	(6)	0.5–29 (± 5.5)	0 (42p), I (9p)	NR	
Global characteristics of the included studies			Observational trials (3) Cohort (2) Case series (10) Case series (9)	2 (1) 3 (4) 4 (19)	Not available (5) None declared (18) Declared (1)	489	233	Range 36-97		F (182p) Ml (28p)	Caucasian (99p) Asian (58p) Hispanic (1p)		Vulva (167p) Penescrotal (22p) Perianal (8p) Axilla (3p)	Range 0.5-130		0 (220p) I (9p)	Range 5-360	

1: This value refers to the total number of participants. The data relating specifically to the cases treated with imiquimod is not available.
Nº (number), LE (level of evidence), Nº of cases (patient with cutaneous Paget's Disease treated with topical Imiquimod), Md (median), M (mean), SD (standard deviation), F (female), Ml (male), p (patients), NR (no reported).

Table S3: Results for JBI critical appraisal tool for quasi-experimental studies.

i.d.	Author; year of publication	Item 1	Item 2	Item 3	Item 4	Item 5	Item 6	Item 7	Item 8	Item 9
16	Cowan, R.A.; 2016	+	+	X	X	X	+	+	+	-
18	Sawada, M.; 2017	+	+	X	X	X	+	+	+	-
20	Van der Linden, M.; 2018	+	+	X	X	X	+	+	-	+
23	Van der Linden; 2022	+	+	X	X	X	+	+	+	+
24	Borella, F.; 2022	+	+	X	X	X	+	+	+	+

Answers to the JBI critical appraisal checklist: Yes ("+"in green circle), No ("x" in red circle), Unclear/ incomplete information ("-" in yellow circle) or Not/Applicable (NA).

Table S4: Results for JBI critical appraisal tool for case series studies.

i.d.	Author; year of publication	Item 1	Item 2	Item 3	Item 4	Item 5	Item 6	Item 7	Item 8	Item 9	Item 10
5	Tanaka, V.D.A.; 2009	x	-	+	+	+	+	+	+	NA	x
6	Pang, J.; 2010	x	+	+	+	+	-	-	+	NA	x
9	Baiocchi, C.; 2012	+	+	+	+	+	+	+	-	NA	x
11	Choi, J.H.; 2013	x	+	+	+	-	+	+	+	NA	x
12	Sanderson, P.; 2013	+	+	+	x	+	-	+	+	NA	x
14	Luyten, A.; 2014	+	+	+	+	+	-	+	x	NA	+
15	Marchitelli, C.; 2014	+	+	+	x	x	-	+	+	NA	x
17	Liau, M.M.; 2016	+	+	+	+	+	+	+	-	NA	x
21	Choi, S.; 2020	+	+	+	+	+	-	-	-	NA	+
22	Christodoulidou, M.; 2021	+	+	+	+	+	-	-	-	NA	x

Answers to the JBI critical appraisal checklist: Yes ("+"in green circle), No ("x" in red circle), Unclear/ incomplete information ("-" in yellow circle) or Not/Applicable (NA).

Table S5: Results for JBI critical appraisal tool for case report studies.

i.d.	Author; year of publication	Item 1	Item 2	Item 3	Item 4	Item 5	Item 6	Item 7	Item 8
1	Zampogna, J.C.	+	+	+	+	+	+	+	+
2	Mirer, E	+	+	+	+	+	+	+	+
3	Hatch, K.D.	+	+	+	+	+	x	+	+
4	Challenor, R.	+	+	+	+	+	+	x	+
7	Sendagorta, E.	+	+	+	+	+	+	+	+
8	Shelbi, C.J.O.	+	+	+	+	+	x	+	+
10	Boulard, C.	+	+	+	+	+	+	+	+
13	Jing, W.	+	+	+	+	+	x	+	+
19	Apalla, Z.	+	+	+	+	+	+	+	+

Answers to the JBI critical appraisal checklist: Yes ("+"in green circle), No ("x" in red circle), Unclear/ incomplete information ("-" in yellow circle) or Not/Applicable (NA).
